# Pectus excavatum with brugada phenocopy electrocardiogram

**DOI:** 10.11604/pamj.2021.38.347.28813

**Published:** 2021-04-12

**Authors:** Ng Kian Seng, Sarvesh Seger

**Affiliations:** 1 Department of Internal Medicine, International Medical University, Clinical Campus Kluang, Kluang, Johor, Malaysia

**Keywords:** Pectus excavatum, brugada phenocopy, electrocardiogram

## Image in medicine

A 17-year-old male consulted us because he had a “bizarre ECG”. The patient was asymptomatic and the electrocardiogram (ECG) was part of a medical examination. Patient had no history of nonvasovagal syncope. There was no history of sudden cardiac death or Brugada syndrome in family members. Examination revealed a prominent pectus excavatum and there was no other skeletal or connective tissue abnormality. The chest radiograph was consistent with the diagnosis of pectus excavatum. The ECG showed deep negative P-wave in lead V1. The rSR' pattern (a partial right bundle branch block) in leads V1, V2 is followed by a rectilinear ST segment which slopes down into an inverted T-wave. Leads V 1-2 are reminiscent of the rectilinear type of Brugada syndrome. V3 does not have a partial right bundle branch block (RBBB) morphology; the coved ST terminates in a T-wave inversion (TWI). Brugada phenocopies are clinical entities characterized by an ECG pattern that is very similar to true Brugada syndrome but are elicited by other conditions. Such entities include electrolyte abnormalities, myocardial ischemia, pericarditis, myocarditis and pulmonary embolism. The Brugada phenocopy ECG pattern resolves after correction or amelioration of the underlying condition. It is believed that mechanical mediastinal compression from pectus excavatum onto the right ventricle causes changes in the right ventricular outflow tract (RVOT) resulting in the ECG abnormalities that mimic a Brugada syndrome.

**Figure 1:**
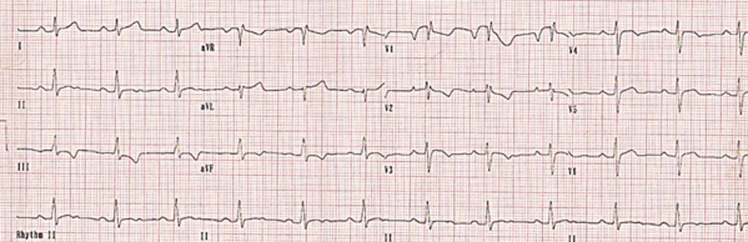
electrocardiogram showed in V1 V2, a rSR morphology, a rectilinear ST that terminates in a TW inversion; diagnosis: brugada phenocopy in a patient with pectus excavatum

